# Oral health and healthy aging: A multiregional review

**DOI:** 10.1016/j.jarlif.2025.100057

**Published:** 2025-12-22

**Authors:** Fatimah Maria Tadjoedin, Melissa Adiatman, Yun Yee Amber Lee, Ummus Sajidah Banu, Sheryl S.L. Tan, Vandana Garg

**Affiliations:** a Dept. Periodontology, Faculty of Dentistry, Universitas Indonesia, Indonesia, Jalan Salemba No. 4, Jakarta 10430, Indonesia; b Dept. Preventive and Public Health Dentistry, Faculty of Dentistry, Universitas Indonesia, Jalan Salemba No. 4, Jakarta 10430, Indonesia; c Haleon, Lot 89, Jalan Enggang, Malaysia; d Haleon, 23 Rochester Park, Singapore

**Keywords:** Healthspan, Healthy aging, Lifespan, Oral diseases, Oral health, Quality of life

## Abstract

Oral health is a critical determinant of overall well-being and healthy aging, especially in countries with growing older populations and health disparities. Maintaining healthy teeth, gums, and orofacial structures improves the quality of life (QoL) while simultaneously reducing the risk associated with several non-communicable diseases through modifying shared risk factors and controlling inflammation. Oral diseases, such as dental caries and periodontal issues, affect nearly 903 million people in Southeast Asia (SEA), with a 61.4% increase in their prevalence from 1990 to 2019. Poor oral health, especially in older individuals, is associated with functional impairments, nutritional deficiencies, psychosocial challenges, and systemic health issues such as diabetes and cardiovascular diseases. Shared risk factors, including dietary habits, stress, and socioeconomic inequalities, compound these challenges. Functional limitations due to oral health inadequacies, such as edentulism, difficulty chewing, pain during eating, and speech impairments, negatively impact nutritional intake and social participation. The impact of oral diseases on QoL and their association with systemic health emphasize the need for preventive strategies and early interventions. Enhancing oral health can bridge the gap between lifespan and healthspan, thereby improving an individual’s QoL, reducing healthcare costs, easing the burden on the healthcare system, and alleviating societal burdens for future generations. Most oral health issues can be managed and mitigated through integrated healthcare strategies, preventive interventions, and public education campaigns. This review emphasizes the need for awareness and a collaborative, interprofessional approach within the healthcare system to ensure equitable access to oral care and support healthy aging across SEA.

## 1 Introduction

Oral health is a fundamental component of an individual’s overall well-being, influencing nutrition, speech, systemic health, and quality of life (QoL). As a key factor in healthy aging, it affects physical, psychological, and functional abilities. Despite being preventable, oral diseases remain stubbornly prevalent worldwide and pose a significant global health challenge, affecting nearly half of the world’s population [1]. The two most common oral diseases—dental caries and severe periodontitis—affect 3.64 billion people, exceeding the combined prevalence of major non-communicable diseases (NCDs), including mental disorders, cardiovascular diseases (CVDs), diabetes mellitus (DM), cancers, and chronic respiratory diseases, at 2.49 billion [2]. Most countries in Southeast Asia (SEA), including Brunei [3], Indonesia [4], Malaysia [5], Singapore [6], Thailand [7], the Philippines [8], and Vietnam [9] carry a considerable burden of oral diseases. Although a non-SEA neighboring country, this burden is also evident in Taiwan [10]. Most people in SEA are impacted by dental caries, dentine hypersensitivity (DH), and periodontal diseases (gingivitis and periodontitis) involving gum tissues, alveolar bone, cementum, and periodontal ligament.

Global projections suggest that by 2050, 2.1 billion people will be over 60 years old, and 426 million will be over 80 years old [11]. In SEA, the older population (≥60 years) is expected to rise from 9.8 % in 2017 to 20.3 % by 2050 [12]. With increasing life expectancy, more people live with chronic NCDs such as diabetes, CVDs, mental health issues (such as dementia), and functional disabilities [13]. Oral diseases and NCDs share common risk factors and have a bidirectional relationship. Poor oral health can worsen functional impairments, nutritional deficiencies, comorbidities, and systemic diseases. Conversely, chronic stress and poor dietary choices are linked to periodontal diseases [[14], [15], [16], [17]]. In older individuals, oral health is further compromised by socioeconomic inequalities, including low income, limited awareness of oral health, and inadequate social support [18]. Retirees with restricted income and lower socioeconomic status (SES) often struggle to access dental care, leading to infrequent dental visits, exacerbated oral issues, and inadequate oral hygiene [19,20]. In Thailand and Indonesia, limited access to dental care and fewer dental visits correlate with higher edentulism rates (up to 11.8 %), especially among lower SES groups [21,22]. Misconceptions, such as believing tooth loss and poor oral health are normal in aging, perpetuate poor oral health practices and reduce QoL in these individuals [23].

Many countries in SEA struggle to provide adequate dental care due to a shortage of dental health professionals, interpersonal communication challenges, the complexity of diseases and polypharmacy, and insufficient integration of oral healthcare into primary healthcare systems. These barriers affect the quality of care provided to patients [24]. Moreover, there is a paucity of region-specific research on oral health-related issues and strategies for mitigating them. This review explores the critical link between oral health and healthy aging in SEA, emphasizing its economic burden and associated challenges. It also focuses on strategies to increase oral health awareness among the public and how a collaborative interprofessional approach within the healthcare system can improve the overall QoL of the older population in these countries.

## 2 Literature review strategy

**Fig. 1:**
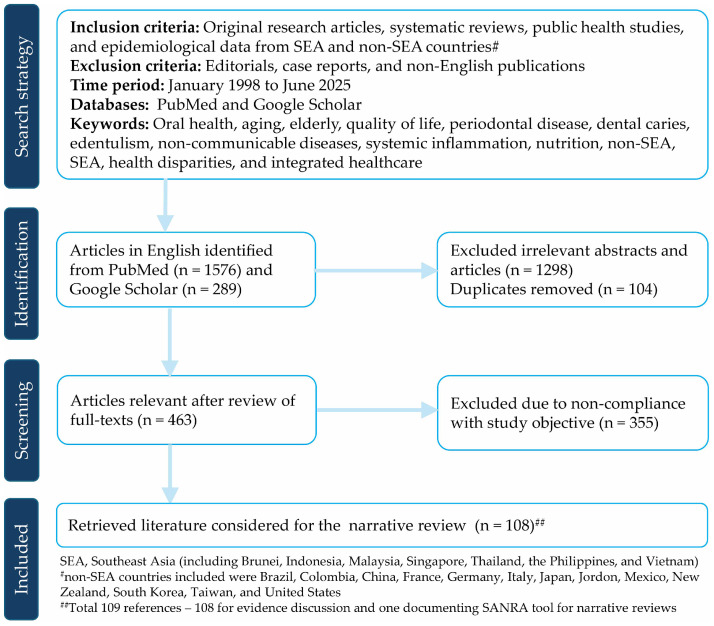
Flowchart of the literature search and evidence selection for the narrative review.

A comprehensive literature search was conducted in PubMed and Google Scholar to identify relevant scholarly articles published in English between January 1998 and June 2025. The search strategy combined Medical Subject Headings and keywords such as “aging,” “dental caries,” “edentulism,” “elderly,” “health disparities,” “integrated healthcare,” “non-communicable diseases,” “non-SEA countries (such as Brazil, China, Colombia, France, Germany, Italy, Japan, Jordan, New Zealand, Mexico, South Korea, Taiwan, and the United States),” “nutrition,” “oral health,” “periodontal disease,” “quality of life,” “Southeast Asia,” and “systemic inflammation” using appropriate Boolean operators (AND, OR). The inclusion criteria encompassed original research articles, systematic reviews, public health studies, and epidemiologic data. Editorials, case reports, and non-English publications were excluded from the narrative review (Fig. 1). This review was conducted in accordance with the Scale for the Assessment of Narrative Review Articles (SANRA), a validated tool to assess the quality and transparency of narrative review articles [25].

## 3 Economic burden and inadequacies in oral health

The economic impact of oral diseases is significant, estimated at USD 710 billion globally in 2019, including USD 387 billion in direct treatment expenses and USD 323 billion in indirect costs, such as lost wages and productivity, highlighting the dual financial impact on healthcare systems and workforce efficiency [1,26]. The global annual costs associated with some of the oral diseases are staggering, amounting to USD 357 billion for treatment and USD 188 billion in productivity losses [27]. Adults lose 3.1 billion work hours yearly due to tooth decay, costing USD 34.7 billion across 40 countries [26]. Poor oral health disproportionately impacts lower-income groups due to high dental care costs, limited access to services, and unhealthy diets. Improving oral health for all could save USD 7.4 billion in Indonesia [28], USD 4.7 billion in the Philippines [29], USD 1.7 billion in Thailand [30] and USD 1.5 billion in Vietnam [31] in lifetime tooth decay costs.

The implications of poor oral health extend beyond the mouth. Oral diseases are also linked to multiple NCDs and systemic diseases. A bidirectional association exists between gum disease and type 2 diabetes (T2D): poorly controlled diabetes raises the risk of periodontitis (severe gum disease), whereas chronic periodontitis worsens glycemic control in individuals with T2D [32,33]. Among people with gum disease, diabetes-related healthcare costs account for USD 1 trillion per decade [26]. Effective oral hygiene and professional care could prevent 57 million cases of T2D globally, unlocking USD 181 billion in economic gains over 10 years [26]. This could save USD 1.5 billion in Indonesia [28], USD 169 million in the Philippines [29], USD 882 million in Thailand [30], and USD 294 million in Vietnam [31] over 10 years in T2D-associated costs.

Access to dental services is limited in most SEA regions, particularly in rural/remote areas, due to inadequate dental infrastructure (clinics, equipment, and supplies) [34]. These gaps significantly hinder the timely diagnosis and treatment of oral health conditions, especially among older individuals.

## 4 Healthy aging—mouth, mind, and body connections

The World Health Organization (WHO) defines healthy aging as “the process of developing and maintaining the functional ability that enables well-being in older age” [35]. It involves creating environments and opportunities that enable people to maintain their health and functional ability. These abilities transcend beyond mental and physical capacities to include home, community, and society for elderlies [36]. Oral health, a key component of overall well-being, is defined by the WHO as “the state of the mouth, teeth and orofacial structures that enables individuals to perform essential functions such as eating, breathing and speaking, and encompasses psychological dimensions such as self-confidence, well-being and the ability to socialize and work without pain, discomfort and embarrassment” [37]. As oral health status changes throughout life, it significantly contributes to healthy aging. Achieving this requires moving beyond a disease-focused model to one that prioritizes prevention, QoL, and holistic well-being.

### 4.1 Lifespan versus healthspan

Lifespan refers to the total number of years a person lives, whereas healthspan refers to the period of life spent in good health, free from chronic disease or disability. Strategies to extend healthspan aim to delay the onset of age-related diseases and disabilities, ensuring longevity with well-being and independence, contributing to community and familial dynamics [38]. This aligns with healthy life expectancy (HALE), which estimates the average number of years an individual can expect to live in full health, without disease or disability [39].

**Fig. 2:**
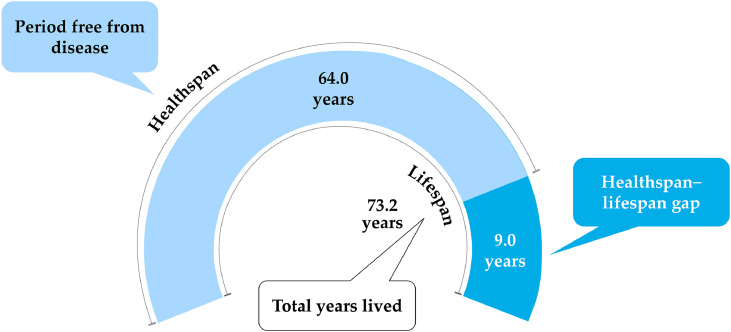
Lifespan versus healthspan. Based on global mean population statistics from 183 WHO member states, the healthspan–lifespan gap is estimated to be approximately 9 years (mean health-adjusted life expectancy = 63.3 years; mean life expectancy = 72.5 years). Improving healthspan encourages healthy aging and eases the burden on healthcare systems. Adapted from: Garmany *et al*., 2024 [41].

Bridging the lifespan–healthspan gap, or the years an individual spends in poor health, is a critical challenge. Globally, it accounts for nearly one-fifth of a person’s life, with many individuals living approximately 9 years with morbidity from one or more chronic NCDs (Fig. 2) [40,41]. It is alarming that the lifespan–healthspan gap is increasing in most SEA countries; in Singapore, the average lifespan is around 84 years, whereas the average healthspan is 74 years (a 10-year gap) [42]. While extending lifespan is important, extending healthspan requires a focus on preventing age-related health issues and maintaining functional ability, with oral health playing a critical role.

### 4.2 Role of oral health in improving healthspan

Healthy aging is not solely about addressing health issues in older adults; it extends to proactive prevention of oral health problems. Poor oral health increases the lifespan–healthspan gap. Hence, maintaining oral health and associated functional abilities is crucial for enhancing lifespan and supporting overall well-being [43,44]. Preventive measures, such as good oral hygiene, a healthy diet, avoiding tobacco and excessive alcohol consumption, play a huge role in maintaining functional abilities throughout life.

Retaining functional teeth and gum health is critical for overall well-being. Oral diseases, including periodontal disease, dental caries, edentulism, DH, tooth erosion, and xerostomia (dry mouth due to reduced saliva production), can lead to reduced QoL and increased dependence or disability. Periodontal disease and edentulism have been linked to higher mortality risk in older individuals, systemic diseases, nutritional deficiencies, and psychosocial challenges [45]. Maintaining good oral health from childhood is essential, as poor oral health in children exacerbates health complications during adulthood. Children with high caries at age 5 were associated with an increased likelihood of certain metabolic issues, such as obesity (body mass index [BMI] ≥30), elevated waist circumference, and higher serum leptin levels. These children also showed signs of accelerated biological aging by age 45, compared to those who were caries-free [46]. Older individuals retaining ≥20 natural teeth experience better aging outcomes, including fewer disabilities, less depression, and greater life satisfaction [47].

Launched in 1989, Japan’s 8020 campaign encouraged people to retain at least 20 natural teeth by 80 years of age, demonstrating the impact of improved oral health on enhancing healthspan [35]. This initiative promoted lifelong oral care through regular dental visits, good hygiene, and a balanced diet. As a result, functional dentition (FD) among 80-year-olds improved from 7 % in 1989 to over 50 % in 2019. It also raised public awareness of oral health and reinforced the importance of preventing tooth loss to prevent NCDs and frailty, promoting overall well-being [35].

## 5 Challenges associated with oral health and healthy aging

Oral health issues are becoming predominant among older individuals due to limited awareness, economic barriers, inadequate access to care, and the perception that oral health decline is inevitable with ageing, deterring many from seeking dental treatment. Ethnic disparities, fear of dental procedures, and negative attitudes toward dental care further worsen oral health outcomes. Migrants from SEA countries present notable challenges in dental healthcare, as their treatment-seeking behaviors are shaped by their cultural background and prior experiences [16]. Consequently, edentulism and periodontitis represent significant complications among older people, causing impaired mastication, poor nutritional choices, speech difficulties, and psychological distress. These conditions are further linked to systemic diseases, including CVD and DM [2]. Globally, the main challenges of poor oral health in older individuals include functional impairments, lifestyle-related challenges, nutritional deficiencies, systemic diseases, and psychosocial concerns.

### 5.1 Functional impairments

Poor oral health and edentulism are frequently associated with functional impairments, particularly in individuals >65 years [48]. Edentulism impairs mastication and chewing, potentially causing swallowing difficulties, leading to a restricted diet, reduced food variety, and a higher risk of nutritional deficiencies. The number of teeth retained affects biting force and denture use, further influencing food intake. Chewing problems are exacerbated by pain and discomfort, particularly in older individuals with ill-fitting dentures or exposed tooth roots, making mealtimes distressing [48]. Another significant challenge is speech impairment, as edentulism affects pronunciation and clarity, causing communication difficulties and social withdrawal [49,50]. A Malaysian study demonstrated that individuals with <20 teeth struggle to chew fiber-rich food and raw green vegetables, and are more likely to avoid them, resulting in the risk of malnutrition [51,52]. According to the Malaysian National Oral Health Survey of Adults 2020 report, 13.9 % of individuals reported oral functional limitations, with 23.8 % reporting discomfort from food stuck between teeth/dentures, and 8.3 % reporting difficulty chewing hard foods [53]. While primarily affecting the oral cavity, these limitations also impact nutritional intake, social interactions, and psychological well-being.

### 5.2 Lifestyle-related challenges

**Fig. 3:**
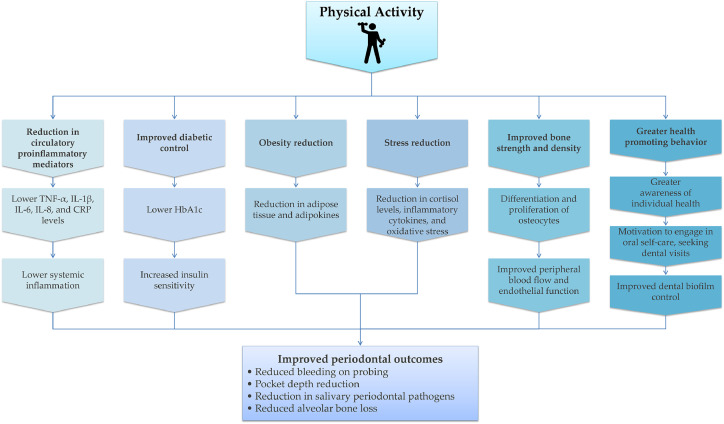
The relationship between physical activity and periodontal disease. Physical activity may directly lower the risk of periodontitis by reducing the circulatory proinflammatory mediators associated with periodontal tissue attachment loss and bone resorption. It can also indirectly protect against periodontitis by reducing risk factors such as DM and combating obesity by increasing energy expenditure, reducing adipose tissue mass, and lowering the level of adipokines. Another mechanism is the stress-reducing effect of physical activity, leading to lower cortisol and inflammatory cytokine levels. Lastly, individuals who engage in regular physical activity may be motivated to adopt health-promoting behaviors, such as consistent oral hygiene practices and seeking regular dental care. Adapted from: Chan et al.*,* 2023 [54]. Abbreviations: CRP: C-reactive protein; HbA1c: Glycated hemoglobin; IL: interleukin; IL-1β: interleukin 1 beta; TNF-α: tumor necrosis factor alpha.

**Fig. 4:**
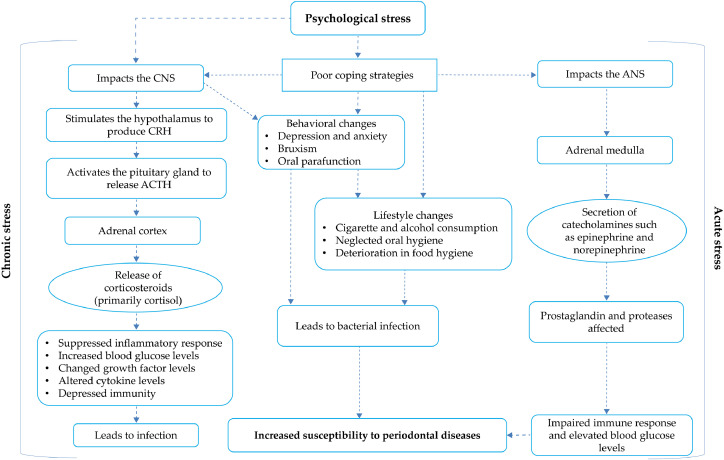
Biological mechanisms and behaviors link psychological stress and periodontal disease. Chronic stress negatively impacts periodontal disease indirectly through behavioral and lifestyle changes, as well as directly through its effect on the neuro-immuno-endocrinological processes. Stress activates the hypothalamus, leading to the release of corticotropin-releasing hormone, which stimulates the pituitary gland to release adrenocorticotropic hormone and trigger cortisol secretion. Acute stress activates the autonomic nervous system and stimulates the adrenal medulla to release catecholamines such as epinephrine and norepinephrine. These hormones affect prostaglandins and proteases, impair the immune response, and increase susceptibility to periodontal destruction over time. Adapted from Gunepin *et al*., 2018 [17] and Genco et al., 1998 [63]. Abbreviations: ANS, autonomic nervous system; ACTH, adrenocorticotropic hormone; CNS, central nervous system; CRH, corticotropin-releasing hormone. Bruxism is a repetitive jaw-muscle activity involving clenching or grinding teeth during sleep or wakefulness.

Aging adults face lifestyle challenges, such as inadequate physical activity, stress, smoking, and poor diet, which affect their general and oral health [54]. Physical activity may benefit periodontitis directly by reducing inflammation and indirectly through modulatory effects on insulin sensitivity, obesity, bone density, and stress regulation (Fig. 3). High physical activity has been significantly associated with a lower prevalence of stage III and IV periodontitis, lower gingival inflammation, probing depths, and fewer sites with clinical attachment loss ≥3 mm [[55], [56], [57]]. A South Korean study reported that regular walking, defined as 30 min per session, five or more days per week (equivalent to ≥150 min of moderate activity per week), significantly reduced the risk of periodontal disease, suggesting that even low-intensity physical activity may reduce the risk of periodontitis [58]. Physical activity is associated with a significant reduction in bleeding on probing, periodontitis severity, and HbA1c levels in patients with diabetes [59]. Consistent with these findings, structured exercise interventions such as strength-endurance, endurance (30-minute treadmill or cycling sessions), or combined training performed two or more times weekly have demonstrated additional oral and systemic health benefits when maintained regularly. Associated with shared pathways of chronic low-grade inflammation and immune cell dysregulation, periodontitis has shown a significant association with BMI, and research suggests that individuals who are lean and physically fit have a reduced risk of severe periodontitis [60,61]. A Japanese study among obese men found regular exercise significantly reduced the number of gum pockets ≥4 mm, the number of teeth with bleeding on probing, and salivary counts of periodontal pathogens [62]. It also reported a positive correlation between regular exercise and levels of periodontal pathogens, body weight, and fasting insulin, supporting a shared inflammatory mechanism between physical activity, periodontitis, and metabolic disorders. Biological mechanisms and behaviors link psychological stress and periodontal disease. Chronic stress worsens periodontal disease indirectly through behavioral and lifestyle changes, and directly via neuro-immuno-endocrinological effects. Increased stress activates the hypothalamic–pituitary–adrenal axis, higher cortisol levels, proinflammatory cytokines, and oxidative stress. Acute stress activates the autonomic nervous system and stimulates the adrenal medulla to release catecholamines (epinephrine and norepinephrine). These hormones affect prostaglandins and proteases, impair the immune response, and increase susceptibility to periodontal destruction over time [17,63] (Fig. 4).

Another well-established risk factor for poor oral health is smoking; smoking severely impairs gum health, hinders tissue repair, and increases the risk of oral cancers [64]. It reduces saliva production, which is essential for neutralizing acids and preventing infections in the mouth. It also impairs wound healing and diminishes immune responses, exacerbating periodontal disease [65]. Consistent with this, a higher prevalence of periodontal disease was noted among Malay older adults who were smokers, with 27 % of patients reporting tobacco use [65].

Dietary habits also critically influence oral health. A high-sugar, high-acid diet accelerates enamel erosion, promoting tooth decay and cavities. Frequent acidic food consumption lowers oral pH, favoring dental caries [66]. Evidence suggests high-glycemic food consumption can increase gingival and periodontal inflammation and gingival bleeding, whereas a diet rich in complex carbohydrates, without increasing total energy intake, may reduce gingivitis and periodontitis risk [[67], [68], [69]]. Combined with regular exercise, a healthy diet, specifically whole grains, vegetables (raw or cooked), fresh fruits, dairy, olive oil, fish, red or white wine, pulses, red meat, dried fruits, while limiting carbonated beverages, butter, cooking cream, sweets or pastries, can reduce the odds of severe periodontitis [55].

### 5.3 Nutritional deficiencies

**Fig. 5:**
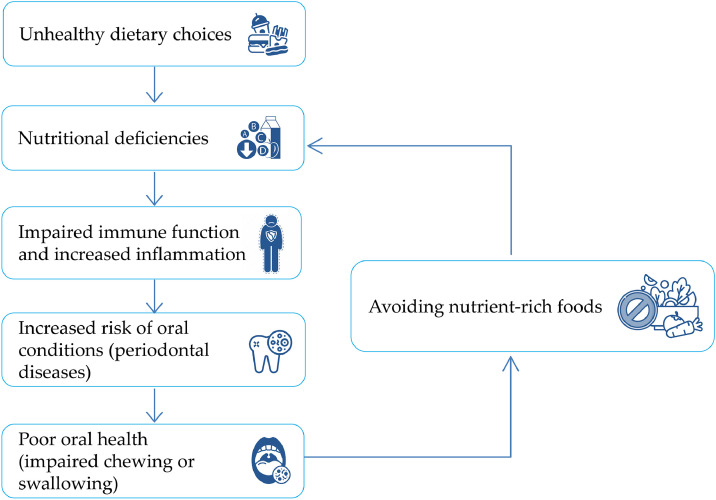
Bidirectional relationship between nutritional deficiencies and oral health. Unhealthy dietary habits lead to inadequate food intake, which is associated with malnutrition. These deficiencies, in turn, impair immune function, increase inflammation, and heighten the risk of oral conditions, such as periodontal diseases. Conversely, poor oral health can hinder the ability to chew and swallow, leading to the avoidance of nutrient-rich foods, exacerbating nutritional deficiencies, and further increasing the risk of oral diseases. Adapted from Denica *et al*., 2021 [71].

Nutrition and oral health exhibit a bidirectional relationship, especially when older people are predisposed to nutritional deficiencies from reduced masticatory and food choices [70]. Poor oral health is often associated with malnutrition and low BMI, perpetuating a negative cycle of oral diseases, malnutrition, and weakened immune function [71] (Fig. 5). Tooth loss is associated with malnutrition, especially in the older population [72]. Compromised dentition affects food intake, causing a 20 % reduction in energy, along with decreases in protein (5 %), dietary fiber (25 %), vitamin A (12 %), and vitamin C (14 %) [73]. A prospective Thai study indicated that older individuals (≥60 years) with FD had higher vegetable intake and healthier dietary patterns than those without FD or dentures [74]. Similarly, the loss of posterior occluding teeth on both sides is associated with reduced consumption of meat, nuts, eggs, fish, dairy products, inadequate protein (<0.8 g/kg body weight), iron, and vitamin B12, and lowered muscle mass indices [75].

Conversely, nutritional deficiencies impair immunity, exacerbate inflammation, and increase the risk of periodontal diseases. A proinflammatory diet and poor micronutrient intake, especially deficiencies in vitamin C, vitamin D, and vitamin B complex, have been linked to higher periodontal disease risk [70]. In Malaysia, around 90 % of adults with signs of periodontal disease had insufficient nutritional intake [65]. Another Malaysian study conducted in 2299 older individuals (≥60 years) reported a significant inadequate intake of magnesium (100.0 %), manganese (97.9 %), zinc (95.6 %), vitamin B6 (98.4 %), potassium (91.0 %), calcium (89.3 %), vitamin B12 (80.2 %), vitamin E (91.2 %) and vitamin K (81.5 %) [76]. It has also been observed that vitamin D enhances the absorption of calcium and magnesium; hence, its supplementation may improve postsurgical periodontal healing. Higher intakes of vitamins A, B, C, and E, along with omega-3 fatty acids, are reported to improve periodontal health upon supplementation [70].

### 5.4 Systemic diseases

The association between oral and systemic health is bidirectional—poor oral health can exacerbate systemic conditions, while underlying diseases can worsen oral health outcomes. A nationwide Chinese study in adults linked tooth loss with mortality, reporting that individuals who lost more than the age-specific median number of teeth had a 35 % increased mortality risk from upper GI cancer, 28 % from heart disease, and 12 % from stroke [77]. Similarly, data from the Taiwan National Health Insurance Research Database indicated that periodontal disease may cause chronic systemic inflammatory diseases, such as chronic obstructive pulmonary disease, chronic kidney disease, rheumatoid arthritis, cognitive impairment, obesity, and metabolic syndrome [78]. This is primarily driven by upregulation of proinflammatory mediators triggered by oral bacterial biofilms and their products, or infiltration of periodontal bacteria and bacterial molecules into the target organs via the bloodstream [78].

DM is one of the most well-documented chronic NCDs affected by oral health. A retrospective study in Malaysia found that 15 % of older people with periodontal disease had DM [65]. Similarly, another study showed that 17.5 % of older Filipino patients with diabetes were more susceptible to periodontal disease [79]. Individuals with severe periodontal disease experience a five-fold increase in HbA1c over 5 years, complicating their diabetes management [80]. Furthermore, periodontal issues are also known to escalate the risk of metabolic syndrome by 2.31 times [81].

Similarly, most CVDs, including hypertension, heart failure, ischemic heart disease, stroke, and coronary heart disease, show a strong correlation with oral health. It has been reported that 31.8 % of older adults with periodontal disease have hypertension [65]. Periodontal disease is associated with a 19 % higher risk of developing CVD, increasing to 44 % in individuals ≥65 years [82]. Chronic oral infections contribute to endothelial dysfunction and arterial plaque formation, heightening the risk of ischemic heart disease and stroke [83]. Notably, periodontal therapy has been shown to reduce systemic inflammation and improve vascular health [84].

Respiratory conditions such as asthma and pneumonia have also been associated with periodontal disease [85]. A Malaysian retrospective study of subjects aged over 50 revealed that asthmatic individuals had mild periodontitis, suggesting a possible link between asthma and periodontal disease due to its chronic inflammatory nature. The use of inhalers (commonly used in asthma) can decrease salivary flow and lead to xerostomia, which may make these patients vulnerable to periodontal issues [65]. Oral bacteria associated with poor oral hygiene can be aspirated into the lungs, leading to infections such as pneumonia. Respiratory illnesses like the flu or pneumonia can alter the composition of the oral microbiome [86].

Osteoporosis, characterized by reduced bone density and increased fracture risk, has significant implications for oral health. Bone loss is often mirrored in the jawbone, leading to alveolar bone resorption, edentulism, and poor denture retention [87]. Research suggests that osteoporotic individuals are more prone to periodontal disease due to decreased bone regeneration capacity and altered inflammatory responses [88,89]. Consistently, a nationwide study on 10,102 Taiwanese individuals indicated osteoporosis as a risk factor for periodontitis [90]. Additionally, medications such as bisphosphonates, commonly used to manage osteoporosis, have been linked to jaw osteonecrosis, further complicating oral health [91].

### 5.5 Psychosocial challenges

Psychosocial factors significantly impact healthy aging and oral health [92]. Older individuals with depression, or those with loneliness and social isolation, often report poor oral health-related QoL [93,94]. Furthermore, poor oral health, especially periodontal issues and xerostomia, negatively impacts the QoL of older adults with reduced Geriatric Oral Health Assessment Index (GOHAI) scores. Noticeable dental problems or oral malodor can cause social withdrawal and lower self-confidence, further declining GOHAI scores [95]. Both edentulism and periodontal diseases are associated with decreased self-esteem, social isolation, psychological distress, loneliness, and low life satisfaction in older adults, thus increasing their emotional burden and leading to neglected oral hygiene [73,94]. A Taiwanese study found that those who experienced a rapid loss of teeth were at a higher risk (1.7 times) of developing psychological distress [96]. Dental pain also increases the risk of depression by 1.59 times and stress by 1.31 times [97]. Moreover, studies have also shown that edentulous older people may experience dementia and poorer cognition compared to those with FD [73,98].

Other conditions, such as DH, substantially impact QoL; pain or discomfort associated with DH may lead to dietary restrictions, promote behavioral changes, and disrupt daily activities, as many sufferers often modify their eating habits and lifestyle to mitigate sensitivity [99]. Additionally, these dental issues impair social interactions and confidence in talking and smiling. Older individuals with limited mobility or reduced access to healthcare often struggle to maintain oral hygiene, leading to higher rates of dental disease [100]. Social engagement supports mental and oral health; older people who participated in community centers and social activities experienced enhanced daily living, fostered social connections, and improved overall well-being [93].

## 6 Strategies to improve oral health for healthy aging

The FDI World Dental Federation asserts that oral health is a basic human right and should be included in universal health coverage [101]. The WHO action plan for oral health in SEA 2022–2030 focuses on “universal coverage for oral health for all people of the SEA by 2030, empowering them to enjoy the highest attainable state of oral health and enabling them to live healthy and productive lives.” Universal coverage can ensure oral healthcare is accessible and affordable to all older adults [34]. In line with this, efforts to save teeth and maintain oral health are crucial for older people. However, maintaining oral hygiene can be challenging for older adults due to age-related factors like reduced physical mobility, cognitive decline, and multiple comorbidities. It is important for all stakeholder policymakers, national dental associations, dentists, and healthcare professionals to recognize the role of oral function in healthy aging and to be aware of their responsibilities in enhancing the oral health of older adults. Achieving this goal requires an integrated approach of oral health support, public education, awareness campaigns, and interventions to promote self-care and compliance with oral hygiene routines.

Educational initiatives have been shown to raise awareness, improve oral hygiene, enhance oral health outcomes, and QoL [102]. Community-based prevention programs, such as regular screenings and mobile clinics, have also demonstrated effectiveness in improving the population’s oral health [103]. Educational programs should promote regular dental visits and oral prophylaxis. Interventional programs that emphasize toothbrushing, oral hygiene, denture care, and oral functional exercises have significantly reduced plaque and gingival scores, enhancing oral health-related QoL in older individuals across SEA [[104], [105], [106], [107]].

The Global Strategy on Oral Health emphasizes that accumulated oral health problems have complex consequences in later life, particularly concerning other NCDs; hence, locally tailored, age-appropriate oral health strategies should be integrated within relevant health programs across the life course [108]. This also calls for a multidisciplinary approach to oral health promotion that involves dental health professionals, allied health professionals, including general practitioners, pharmacists, nurses, and dietitians, and integration of oral health into the overall health management [109].

In developing SEA countries, oral health is often deprioritized due to other health priorities, leaving dental problems untreated, with little emphasis on prevention and limited access to care. Policies should prioritize early preventive measures to decrease the incidence of dental caries, edentulism, and periodontitis, especially among older people.

## 7 Strengths and limitations

This review followed a SANRA-guided narrative approach to synthesize evidence on oral health and healthy aging in SEA. Hence, there is a potential risk of selection bias and heterogeneity in study designs, data sources, and outcome measures. In several domains, SEA-specific evidence remains limited. However, this review provides a consolidated overview of current knowledge and highlights priority areas for future research to better understand the oral health trajectories in aging populations within SEA.

## 8 Conclusion

Oral health is a critical determinant of overall well-being and healthy aging. In SEA, older individuals facing challenges due to dental caries, periodontal issues, DH, and edentulism are known to impair their nutritional status, systemic health, psychological health, and QoL. These challenges are compounded by age-related factors such as cognitive decline, reduced physical activity, and systemic comorbidities such as DM and CVD. Addressing these issues requires integrating oral health into broader health management through a multidisciplinary approach involving dental health professionals, general practitioners, pharmacists, dietitians, national dental associations, and policymakers. Achieving this requires a combined approach to oral health support, public education, awareness campaigns, and interventions that promote prevention, self-care, and adherence to oral hygiene routines.

## Funding

Manuscript development funding was provided by Haleon. The sponsor had no role in the study design, data collection, analysis, interpretation, writing of the manuscript, or decision to submit the article for publication.

## Conflicts of interest

V.G., Y.Y.A.L., U.S.B., and S.S.L.T. are employees of Haleon. F.M.T. and M.A. have no conflicts of interest to declare.

## Data availability

As this is a review article, no new data were generated, and all the information cited was from previously published sources.

## Ethics approval

This review article is based on published literature; hence, ethics approval is not applicable.

## Consent for publication

Not applicable, as this article does not include any individual participant data.

## Declaration of the use of generative AI and AI-assisted technologies in scientific writing and in figures, images, and artwork

None.

## CRediT authorship contribution statement

**Fatimah Maria Tadjoedin:** Writing – review & editing, Writing – original draft, Conceptualization. **Melissa Adiatman:** Writing – review & editing, Conceptualization. **Yun Yee Amber Lee:** Writing – review & editing, Writing – original draft, Conceptualization. **Ummus Sajidah Banu:** Writing – review & editing, Writing – original draft. **Sheryl S.L. Tan:** Writing – review & editing, Conceptualization. **Vandana Garg:** Writing – review & editing, Writing – original draft, Conceptualization.

## Declaration of competing interest

The authors declare the following financial interests/personal relationships which may be considered as potential competing interests: Vandana Garg reports a relationship with Haleon that includes: employment. Yun Yee Amber Lee reports a relationship with Haleon that includes: employment. Ummus Sajidah Banu reports a relationship with Haleon that includes: employment. Sheryl S.L. Tan reports a relationship with Haleon that includes: employment. There are no other activities to disclose. If there are other authors, they declare that they have no known competing financial interests or personal relationships that could have appeared to influence the work reported in this paper.
